# Recent Updates of PET in Lymphoma: FDG and Beyond

**DOI:** 10.3390/biomedicines12112485

**Published:** 2024-10-29

**Authors:** Sung-Yong Kim, Hyun Woo Chung, Young So, Mark Hong Lee, Eun Jeong Lee

**Affiliations:** 1 Division of Hemato-Oncology, Department of Internal Medicine, Konkuk University Medical Center, Konkuk University School of Medicine, 120-1 Neungdong-ro, Gwangjin-gu, Seoul 05030, Republic of Korea; sykim@kuh.ac.kr (S.-Y.K.); mlee@kuh.ac.kr (M.H.L.); 2 Department of Nuclear Medicine, Konkuk University Medical Center, Konkuk University School of Medicine, 120-1 Neungdong-ro, Gwangjin-gu, Seoul 05030, Republic of Korea; youngso@kuh.ac.kr; 3 Research Institute of Medical Science, Konkuk University School of Medicine, 120-1 Neungdong-ro, Gwangjin-gu, Seoul 05030, Republic of Korea; 4 Department of Nuclear Medicine, Seoul Medical Center, 156 Sinnae-ro, Jungnang-gu, Seoul 02053, Republic of Korea; cateunjeong@naver.com

**Keywords:** lymphoma, FDG PET, FAPI, immunoPET, AI

## Abstract

Lymphoma is one of the most common cancers worldwide, categorized into Hodgkin lymphoma and non-Hodgkin lymphoma. ^18^F-fluorodeoxyglucose positron emission tomography (FDG PET) has become an essential imaging tool for evaluating patients with lymphoma in terms of initial diagnosis, staging, prognosis, and treatment response assessment. Recent advancements in imaging technology and methodologies, along with the development of artificial intelligence, have revolutionized the evaluation of complex imaging data, enhancing the diagnostic and predictive power of PET in lymphoma. However, FDG is not cancer-specific, but it primarily reflects glucose metabolism, which has prompted the investigation of alternative PET tracers to address this limitation. Novel PET radiotracers, such as fibroblast activation protein inhibitors targeting the tumor microenvironment, have recently shown promising results in evaluating various malignancies compared to FDG PET. Furthermore, with the rapid advancements in immunotherapy and the favorable imaging properties of ^89^Zr, immunoPET has emerged as a promising modality, offering insights into the functional and molecular status of the immune system. ImmunoPET can also facilitate the development of new antibody therapeutics and radioimmunotherapy by providing pharmacokinetic and pharmacodynamic data. This review provides comprehensive insights into the current clinical applications of FDG PET in lymphoma, while also exploring novel PET imaging radiotracers beyond FDG, discussing their mechanisms of action and potential impact on patient management.

## 1. Introduction

Lymphoma is one of the most common cancers worldwide and is categorized into Hodgkin lymphoma (HL) and non-Hodgkin lymphoma (NHL). HL is a type of B-cell lymphoma, while NHL includes all B-cell lymphomas or natural killer (NK)/T-cell lymphomas other than HL. The major subtypes of NHL include B-cell lymphomas such as diffuse large B-cell lymphoma (DLBCL), follicular lymphoma (FL), marginal zone lymphoma (MZL), and mantle cell lymphoma (MCL) [1].

The traditional Ann Arbor staging classification for lymphoma was originally developed over 50 years ago to determine the extent of radiation therapy for HL when detecting disease outside the radiation field was crucial for treatment decisions [2]. This classification has been useful in describing the anatomical extent of disease in both HL and NHL. In 1989, the Cotswold classification, modified from the Ann Arbor staging system, incorporated computed tomography (CT) for the first time to evaluate intrathoracic and infradiaphragmatic lymph nodes in HL [3]. In 2007, ^18^F-fluorodeoxyglucose positron emission tomography (FDG PET), which assesses glucose metabolism in tissues not clearly characterized by CT alone, was formally introduced in the revised response criteria for malignant lymphoma by the International Working Group [4]. In 2014, the Lugano classification recommended a comprehensive evaluation for both HL and NHL, including history taking, physical examination, complete blood count, lactate dehydrogenase measurement, liver function tests, FDG PET, contrast-enhanced CT, and bone marrow (BM) aspiration and biopsy [5].

FDG PET has become an essential imaging tool for evaluating patients with lymphoma in terms of initial diagnosis, staging, prognostication, and treatment response assessment. Recently, advancements in imaging technology and methodologies, alongside the development of artificial intelligence (AI), have revolutionized the evaluation of complex imaging data. AI can assess large datasets comprehensively in a rapid and precise manner, and its deployment in PET imaging enables faster image interpretation and more accurate diagnosis and risk stratification [6].

However, because FDG is not fundamentally cancer-specific but merely reflects glucose metabolism, alternative PET tracers have been investigated to overcome this limitation. The tumor microenvironment (TME) is generated by interactions between tumor cells and surrounding non-tumorous cells through the circulatory and lymphatic systems, and it plays a crucial role in tumor development, progression, and response to therapies. The TME includes surrounding stromal cells, vascular and immune cells, as well as the extracellular matrix. One of the most prominent stromal cell types in the TME is the cancer-associated fibroblast (CAF). Fibroblast activation protein (FAP), a transmembrane glycoprotein expressed by CAFs, has been suggested as a target for tumor diagnosis and therapy. Recently, novel PET imaging using a radiolabeled FAP inhibitor (FAPI) has shown promising results in evaluating various malignancies, offering potential advantages over FDG PET [7,8].

Immunotherapy has dramatically improved patient outcomes in lymphoma. However, despite rapid advancements in immunotherapy, some patients do not benefit from this treatment, and some may even experience dismal tumor growth, known as hyperprogressive disease. Additionally, conventional imaging methods face new challenges, such as pseudoprogression and immune-related adverse effects associated with immunotherapy [9]. To provide information on the functional and molecular status of the immune system, immunoPET combines the targeting specificity of radiolabeled monoclonal antibodies (mAbs) with the sensitivity of PET imaging. The development of immunoPET has accelerated in recent years, with the recognition of the favorable PET imaging properties of ^89^Zr. With a physical half-life of 3.3 days, ^89^Zr allows PET imaging of large molecules with slow kinetics, such as antibodies, for up to a week using conventional scanners [10]. Radioimmunotherapy (RIT) involves the targeted delivery of therapeutic radionuclides using antibodies. ImmunoPET can also facilitate the development of new antibody therapeutics and RIT by providing pharmacokinetic and pharmacodynamic information, as well as quantifying tumor antigen levels relevant to treatment decisions [11].

This review article provides comprehensive insights into the current clinical applications of FDG PET in lymphoma. Additionally, it explores novel targets for PET imaging beyond FDG, describing their mechanisms of action and potential impact on patient management.

## 2. FDG PET

### 2.1. Initial Diagnosis

A definitive pathological diagnosis and accurate staging at the initial presentation of lymphoma are essential to determine the optimal therapy. Incisional or excisional biopsy is the preferred method for obtaining an adequate specimen for accurate diagnosis, compared to fine-needle aspiration. Core-needle biopsy is considered when excisional biopsy is not feasible [12]. FDG PET can help identify the optimal biopsy site for confirmation by targeting the most active area of a suspected lesion with high sensitivity, compared to CT or ultrasound, particularly in patients with discordant histology or suspected malignant transformation [13]. Broccoli et al. reported that the diagnostic yield of FDG PET-guided needle biopsy in patients with suspected lymphoma was 87.5% (84/96), with a sensitivity, specificity, positive predictive value (PPV), and negative predictive value (NPV) of 96%, 100%, 100%, and 75%, respectively [14].

FDG avidity in the main subtypes of lymphoma is listed in Table 1 [15,16,17,18,19,20,21]. FDG avidity is generally higher in aggressive NHL subtypes compared to indolent NHL subtypes [22]. Highly aggressive NHL subtypes include Burkitt’s lymphoma, B lymphoblastic lymphoma, T lymphoblastic lymphoma, and adult T-cell lymphoma. Aggressive NHL subtypes include DLBCL, grade III FL, certain MCL, peripheral T-cell lymphoma (PTCL), and anaplastic large-cell lymphoma. Indolent NHL subtypes include grade I and II FL, MZL, small lymphocytic lymphoma, lymphoplasmacytic lymphoma, certain MCL, and mycosis fungoides.

### 2.2. Staging

FDG PET has been accepted as a standard imaging modality for initial staging in HL and FDG-avid NHLs. Compared to CT alone, FDG PET often leads to upstaging rather than downstaging by detecting more nodal and/or extranodal lesions with improved accuracy. This is particularly important in patients with early-stage disease and before considering radiation therapy. Metser et al. reported a prospective multicenter study of 850 patients on the management and outcomes of HL or aggressive NHL with apparent limited-stage disease [23]. FDG PET upstaged approximately 18% of patients and changed the planned management in about 39%. Planned therapy was changed in 224 of 580 participants (38.6%) after FDG PET. Furthermore, FDG PET-guided treatment achieved lower 1-year mortality compared to CT-guided treatment alone in NHL (6% vs. 14.2%, respectively). Another large international cohort study of patients with advanced HL (stage IIB-IV and stage IIA with adverse features) reported that FDG PET upstaged 14% of patients (159/1171) and downstaged 6% (74/1171) [24]. Upstaging was primarily due to the detection of extranodal disease in the BM (92 patients), lung (11 patients), or multiple organs (12 patients), with additional nodal disease detected in 35 patients. In a study by Albano et al. of 46 patients with primary brain lymphoma, FDG PET found extracranial disease involvement in 2 patients, leading to changes in clinical management [25]. However, in a study of 95 patients with PTCL, where 96% (91/95) showed high FDG avidity, FDG PET altered the clinical stage in only 5% (5/95) of patients. Staging increased in 2 patients (from stage I to III and from II to IV) and decreased in 3 patients (from stage IV to III, IV to III, and III to 0). Treatment was not changed by the stage adjustment, largely because chemotherapy was administered regardless of the stage [26].

FDG PET is also useful in determining the necessity for blind BM biopsy in patients with HL and DLBCL. A positive FDG PET scan can eliminate the need for a BM biopsy to detect BM involvement in these patients with high certainty. However, a negative FDG PET scan cannot fully rule out BM involvement. FDG PET has been reported to miss 10% to 20% of cases with low-volume diffuse BM involvement in DLBCL [27].

Spleen involvement in lymphoma can result in a spleen that is either normal in size or enlarged. FDG PET assesses spleen involvement more accurately than CT or MRI and may reveal single or multiple FDG-avid lesions or diffuse FDG activity greater than that in the liver and bone marrow. However, false-positive diffuse splenic FDG activity can occur due to variations in blood volume caused by hematopoietic proliferation or the use of hematopoietic stimulating agents [28].

### 2.3. Pretreatment Prognostication

Prognostic biomarkers are defined as objective measurements that provide information on disease outcomes, independent of the treatment received. These biomarkers help identify patients with more aggressive disease as well as those with more indolent forms. Due to the heterogeneity of lymphoid malignancies, determining the appropriate treatment at diagnosis remains challenging for certain tumors, such as FL. The prognosis for many lymphoid malignancies can often only be determined after initial treatment. Therefore, there is a continuing need for rapid and impactful tumor-specific biomarkers across the various subtypes of lymphoma [29].

#### 2.3.1. Standardized Uptake Value

FDG accumulation in tissue is proportional to the level of glucose metabolism. The most widely used quantitative method for measuring tumor metabolic activity via PET is the standardized uptake value (SUV), which is a ratio of the radioactivity concentration in a selected region of interest (ROI) normalized to the injected dose of radioactivity per patient size, such as body weight (SUV), lean body mass (SUL), or body surface area (SUA). This ratio would be one if the radioactivity were distributed homogeneously throughout the body. SUV can be expressed as the radioactivity concentration in the ROI as an average (SUVmean), maximum (SUVmax, hottest voxel), or peak (SUVpeak, which represents the maximum average SUV within a spherical volume of 1 cm^3^). Among these, SUVmax is the most commonly used due to its high reproducibility and ease of calculation [30,31].

A high SUVmax, generally greater than 10, has been suggested as a marker to distinguish between indolent and aggressive NHLs and to predict high-grade transformation from indolent lymphoma [13]. However, a retrospective analysis, which followed 549 patients over five years of treatment, found that baseline SUVmax on FDG PET did not predict the future histological transformation of FL to aggressive lymphoma [32]. The reliability of SUV can be influenced by several factors, such as the time between injection and image acquisition, partial volume effects, and extravasation of the tracer at the injection site. Standardization of data acquisition and reconstruction parameters is also essential for quantitative accuracy [33].

#### 2.3.2. Metabolic Tumor Volume and Total Lesion Glycolysis

According to the Ann Arbor staging system, the presence of a bulky tumor has been recognized as a significant prognostic factor in lymphoma for many years. However, the proposed size thresholds for bulky disease have varied for HL and NHL. In HL, a single mass larger than 10 cm or greater than one-third of the transthoracic diameter at any thoracic vertebra level on a CT image is considered bulky. In NHL, the thresholds are 6 cm for FL and 6 to 10 cm for DLBCL in the rituximab era. Therefore, the Lugano classification recommended recording the largest tumor size measured by CT instead of using the term bulky disease [5].

Not only the presence of a bulky tumor but also a high overall tumor burden has been suggested as an unfavorable prognostic parameter across all lymphoma subtypes. Overall tumor burden can be simply defined as the total amount of cancer in the body, but it cannot be accurately measured by CT alone [34]. Recently, FDG PET/CT has enabled the estimation of overall tumor burden using metabolic tumor volume (MTV) or total lesion glycolysis (TLG). MTV represents the volume of FDG-avid tumor measured by the automatically segmented volume-of-interest (VOI). Total MTV (TMTV) is the sum of all MTVs in a patient’s PET imaging. TLG assesses the degree of glucose accumulation within the tumor VOI and is calculated as MTV × SUVmean. Total TLG (tTLG) is the sum of all TLGs. Various methods have been applied to define the VOI for MTV, such as uptake above the threshold of SUV 4.0 or 2.5, or as a percentage of the SUVmax, typically at 41% or 50%, in each tumor [35].

Mikhaeel et al. reported a study including 147 patients with DLBCL, finding that baseline TMTV, which showed a strong correlation with tTLG, was a significant predictor of progression-free survival (PFS) on multivariate analysis with a median follow-up of 3.8 years [36]. A meta-analysis of 27 studies involving 2729 patients evaluated the prognostic potential of baseline TMTV and tTLG in lymphoma (17 studies on DLBCL, 3 on FL, 1 on PTCL, 4 on extranodal NK/T-cell lymphoma, and 3 on HL) [37]. The analysis found that patients with high baseline TMTV had worse PFS and overall survival (OS) in DLBCL, FL, extranodal NK/T-cell lymphoma, and HL. Similarly, high baseline tTLG was associated with poorer PFS and OS in DLBCL and extranodal NK/T-cell lymphoma. Recently, the Positron Emission Tomography Reanalysis consortium, which included 1214 patients with newly diagnosed DLBCL from five international trials, proposed a new prognostic index called the International Metabolic Prognostic Index [38]. This index, which uses TMTV, age, and stage, outperforms the International Prognostic Index alone. An ad hoc analysis from an international multicenter phase III trial including 406 patients with advanced FL also reported that baseline TMTV was a strong prognostic factor for PFS [39].

Metabolic tumor burden may be more important than anatomical tumor burden for treatment outcomes, particularly in immunotherapy. Chimeric antigen receptor (CAR) T-cell therapy is an emerging treatment for relapsed or refractory lymphoma. However, many patients treated with CAR T-cell therapy experience disease relapse or progression within 6 months. Additionally, life-threatening immune-mediated toxicities, such as cytokine release syndrome (CRS) and neurotoxicity, are common [40]. Dean et al. reported that high baseline TMTV was associated with grade 3–4 CRS (*p* = 0.019) in patients with large B-cell lymphoma who received CAR T-cell therapy. Patients with low baseline TMTV had significantly better PFS (*p* = 0.02) and OS (*p* = 0.005) [41]. Another retrospective study of 180 patients with large B-cell lymphoma found that a higher baseline TMTV (increase of 100 mL) was associated with CRS of grade ≥ 2 (*p* = 0.031) and shorter PFS and OS (*p* < 0.001 for both). Higher baseline SUVmax (increase of 10 units) was associated with failure to achieve complete remission (*p* < 0.001) [42]. However, a recent study by Voltin et al. reported a weak correlation between TMTV and CRS (r = 0.359; *p* = 0.004) and neurotoxicity grade (r = 0.298; *p* = 0.019) in patients with large B-cell lymphoma undergoing CAR T-cell therapy, likely due to the limited number of high-grade toxicities and improved management [43].

#### 2.3.3. Maximum Tumor Dissemination

**Figure 1:**
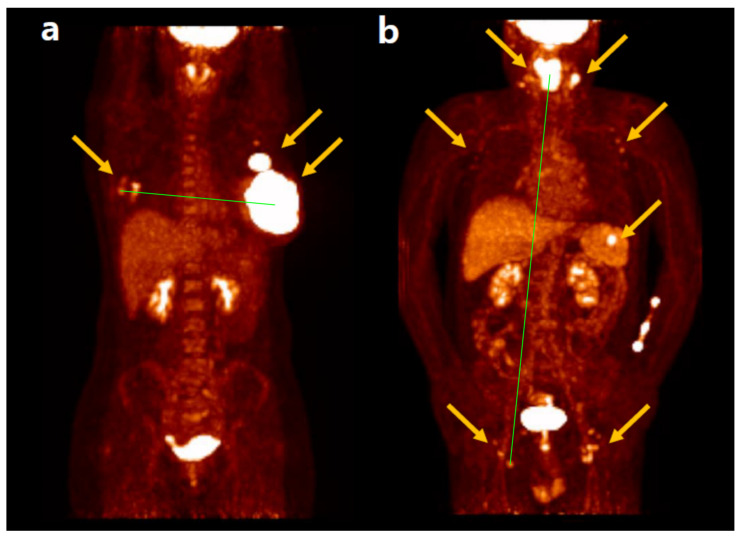
FDG PET/CT images of patients with DLBCL (indicated by arrows) who have known high-risk factors for relapse, in addition to Dmax (line). (**a**) A 38-year-old woman with Ann Arbor stage IV, ECOG PS 0, serum LDH 969 IU/L, IPI score 3, SUVmax 35.4, TMTV 362 mL, tTLG 8287, interim DS 4, EOT DS 4, and Dmax 253.1 mm. No recurrence was found until the last clinical follow-up at 107.7 months. (**b**) A 61-year-old man with Ann Arbor stage III, ECOG PS 1, serum LDH 461 IU/L, IPI score 3, SUVmax 11.4, TMTV 71.8 mL, tTLG 352.5, interim DS 2, EOT DS 1, and Dmax 743.9 mm. Recurrence was found after 16.6 months of follow-up. He survived until the last clinical follow-up at 77.3 months.

The maximum tumor dissemination (Dmax), a concept derived from TMTV, measures the three-dimensional maximum distance between the two FDG-avid lymphoma lesions that are farthest apart. Dmax is a straightforward and intuitive feature to represent tumor burden and dissemination. Because the distance between the center positions of the two tumor lesions is measured automatically after tumor segmentation by software, Dmax is not highly influenced by the operator or tumor contour. Additionally, Dmax can be normalized by body surface area and calculated as standardized Dmax [44]. The significant prognostic role of Dmax in predicting PFS and/or OS has been demonstrated in patients with lymphoma. The most studied lymphoma subtype is DLBCL, followed by HL, with a prevalence of advanced stages (stage III and IV vs. I and II) [45]. Furthermore, Dmax appears to enhance its prognostic value when combined with metabolic variables such as MTV or TLG [46]. Representative cases of Dmax in a patient with DLBCL are shown in Figure 1 [47].

### 2.4. Treatment Response Assessment

**Figure 2:**
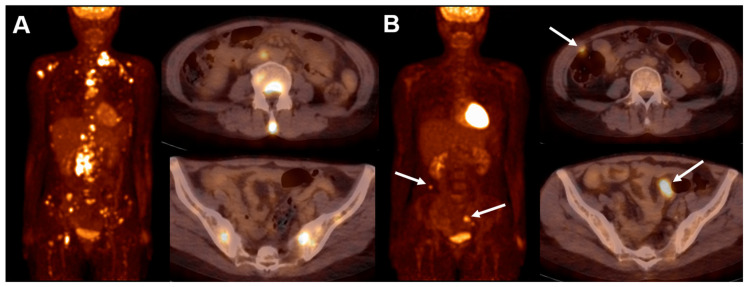
FDG PET/CT images of a 51-year-old woman with DLBCL. She had undergone a total hysterectomy and bilateral salpingectomy for leiomyosarcomona two months prior to the diagnosis of DLBCL. (**A**) Initial maximum-intensity projection and axial images demonstrated multiple areas of FDG uptake in the left supraclavicular, left axillary, bilateral mediastinal, retroperitoneal lymph nodes, as well as in the bones. (**B**) After 3 cycles of R-CHOP, iPET revealed new FDG uptakes in the peritoneum (arrows). Pathological confirmation of metastatic high-grade pleomorphic sarcoma was made through exploratory laparotomy. A complete metabolic response was observed in the initial lymphoma involvements (DS 1).

The standardization of how to report FDG PET results is essential for accurate response assessment. In 2009, the Deauville criteria were defined for simple and reproducible visual interpretation of interim PET (iPET) in HL and DLBCL using a 5-point Deauville score (DS) [48]. The DS is defined as follows: (1) no uptake above background; (2) uptake ≤ mediastinum; (3) uptake > mediastinum but ≤ liver; (4) uptake moderately > liver; (5) uptake markedly higher than liver and/or new lesions; X) new areas of uptake unlikely to be related to lymphoma. The terms “moderately” in score 4 and “markedly” in score 5 suggest that uptake is > SUVmax in a large region of normal liver and 2× to 3× > SUVmax in the liver, respectively. In 2014, the Lugano classification extended the use of the DS from interim to end-of-treatment (EOT) PET [49]. EOT PET assessment by DS improved the NPV of unconfirmed complete remission by CT or partial response of the disease. However, if further treatment is considered based on metabolically active residual disease on EOT PET, either biopsy or follow-up scan is advised due to the relatively lower PPV, particularly in aggressive NHL, which can be caused by inflammation or bone remodeling [50]. Representative case of treatment response assessment using FDG PET is shown in Figure 2.

#### 2.4.1. Response Assessment for Hodgkin Lymphoma

Due to the high curability of early-stage HL, several trials have been conducted to de-escalate the standard 4-cycle chemotherapy and consolidation radiotherapy. The Randomized Phase III Trial to Determine the Role of FDG PET Imaging in Clinical Stages IA/IIA Hodgkin’s Disease (RAPID) clinical trial assessed iPET after three cycles of doxorubicin, bleomycin, vinblastine, and dacarbazine (ABVD) to guide the necessity of consolidation radiotherapy in patients with clinical stage IA or IIA HL without mediastinal bulk disease [51]. Patients with positive iPET (DS ≥ 3 in this study) received a fourth cycle of ABVD and 30 Gy of involved-field radiation therapy (IFRT). Patients with negative iPET (DS 1 or 2) were randomly assigned to receive either IFRT or no further treatment. The 3-year PFS was 94.6% in the IFRT group and 90.8% in the no-further-treatment group. Non-inferior PFS was not observed in the no-further-treatment group compared to the IFRT group.

An international randomized phase III trial with early-stage favorable HL evaluated the role of iPET after 2 cycles of ABVD [52]. In patients with negative iPET (DS 1 or 2), the 5-year PFS was 93.4% for those receiving IFRT and 86.1% for those not receiving IFRT (*p* = 0.04). This result was consistent with the previous RAPID trial. The 5-year OS was not significantly different between the two treatment groups (98.1% vs. 98.4%, respectively; *p* = 0.12). Among patients who underwent 2 cycles of ABVD and IFRT, the 5-year PFS was 88.4% for those with positive iPET and 93.2% for those with negative iPET (*p* = 0.047). When a DS cutoff of 4 was used for positive iPET, the difference in 5-year PFS was more pronounced: 80.9% in positive iPET and 93.1% in negative iPET (*p* < 0.001). However, the OS remained not significantly different.

The H10 Intergroup trial (European Organisation for Research and Treatment of Cancer, Lymphoma Study Association, and Fondazione Italiana Linfomi) provided another analysis of treatment strategies based on iPET response after two cycles of ABVD in stage I and II HL [53]. For patients with positive iPET, the 5-year PFS improved from 77.4% with standard ABVD plus involved-node radiotherapy (INRT) to 90.6% with intensification to escalated doses of bleomycin, etoposide, doxorubicin, cyclophosphamide, vincristine, procarbazine, and prednisone (eBEACOPP) plus INRT (*p* = 0.002). For patients with negative iPET, the 5-year PFS for the favorable risk group was 99.0% vs. 87.1% for ABVD plus INRT and ABVD alone, respectively. The 5-year PFS for the unfavorable risk group was 92.1% vs. 89.6% for ABVD plus INRT and ABVD alone, respectively. For both favorable and unfavorable groups, noninferiority of ABVD alone compared with standard ABVD plus INRT could not be achieved.

Since disease progression necessitates additional treatments, which incur higher costs, PFS is a crucial endpoint for patients. All the studies evaluating the role of iPET in patients with early-stage HL suggest that those with a positive iPET are at high risk for relapse and should be considered for intensified treatment options, such as eBEACOPP combined with IFRT or INRT. In contrast, radiotherapy cannot be omitted from the standard first-line therapy for patients with negative iPET, even if they do not have clinical risk factors.

Recently, a multicenter open-label randomized phase III trial involving patients with early-stage unfavorable HL reported that radiotherapy can be omitted without compromising efficacy in patients who achieve a complete metabolic response by iPET after receiving a two-cycle regimen of eBEACOPP plus two cycles of ABVD (2 + 2 regimen) [54].

A prospective randomized controlled trial, known as the Response-Adjusted Therapy for Advanced HL (RATHL), investigated the use of iPET after two cycles of ABVD to guide treatment for patients with advanced HL (Ann Arbor stage IIB to IV, or stage IIA with adverse features such as bulky disease or at least three involved sites) [55]. Patients with negative iPET (DS 1, 2, or 3) were randomly assigned to continue ABVD (ABVD group) or omit bleomycin (AVD group) in cycles three through six. Patients with positive iPET (DS 4 or 5) received three cycles of eBEACOPP (repeated every 21 days) or four cycles of accelerated BEACOPP (BEACOPP-14, repeated every 14 days). If a second iPET (iPET2) was positive again, radiotherapy or a salvage regimen was administered. If iPET2 was negative, an additional cycle of eBEACOPP or two cycles of BEACOPP-14 were given with no radiotherapy.

The long-term results of the RATHL trial recently reported that patients with a negative iPET can be de-escalated to AVD without inferior outcomes, while those with a positive iPET can be effectively and safely intensified with eBEACOPP/BEACOPP-14 [56]. The 3-year PFS was 85.5% in the ABVD group and 84.3% in the AVD group, which was within the predefined noninferiority margin. The 7-year OS was 93.2% in the ABVD group and 93.5% in the AVD group, with no significant difference between groups. In patients who received eBEACOPP/BEACOPP-14 after a positive iPET, the 7-year PFS was 65.9% and the 7-year OS was 83.2%. The cumulative 7-year incidence of second malignancies was 5.1% for those receiving ABVD/AVD and 2.5% for those receiving eBEACOPP/BEACOPP-14.

Recently, antibody-drug conjugates such as brentuximab vedotin (BV) and immune checkpoint inhibitors (ICIs) such as nivolumab and pembrolizumab, which were originally approved for relapsed or refractory HL, have been incorporated into first-line therapy for advanced HL. Patients who received BV-AVD for the treatment of stage III or IV HL had a survival advantage over those who received ABVD. The 6-year OS estimates were 93.9% in the BV-AVD group and 89.4% in the ABVD group [57]. Moreover, a preliminary report from the Southwest Oncology Group (SWOG) S1826 trial showed improved 1-year PFS with fewer immune-related adverse events after nivolumab-AVD compared to BV-AVD in 994 patients with stage III/IV HL [58]. With increasing evidence supporting BV and PD-1 inhibitors as alternative options, starting treatment with eBEACOPP for advanced HL is less attractive due to its toxicity and the lack of a definitive OS advantage compared to ABVD alone.

#### 2.4.2. Response Assessment for Non-Hodgkin Lymphoma

Although the benefit of de-escalation of treatment using iPET has been well assessed in HL, it has not been clearly demonstrated in NHL. The clinical use of FDG PET varies among DLBCL, PTCL, and FL because the treatment goals for each disease are different. In aggressive lymphomas, the primary role of imaging is to assess remission at the EOT and to identify patients who may require salvage or second-line therapy. In indolent lymphomas, the aim is to control and monitor the disease over several years while maintaining quality of life. Due to the success of the regimen with rituximab, cyclophosphamide, doxorubicin, vincristine, and prednisone (R-CHOP) in patients with DLBCL and the relatively poor prognosis of aggressive T-cell lymphomas despite available treatments, de-escalation of treatment is generally not expected in these circumstances. Even if iPET is positive, many patients with DLBCL who achieve complete remission by R-CHOP at EOT PET have shown a good prognosis, making changes to the R-CHOP regimen based on positive iPET results clinically irrelevant [59]. Similar results are observed in the management of PTCL, and PET-guided treatment in FL has also not demonstrated a significant role.

In patients with aggressive B-cell or T-cell lymphomas (primarily DLBCL), those with positive iPET after 2 cycles of R-CHOP were randomized to receive either an intensive Burkitt-like regimen or to continue with the induction of R-CHOP for an additional four cycles [60]. However, no benefit in PFS or OS was observed from the more aggressive regimen. Furthermore, patients who received the modified therapy experienced increased toxicity.

Rituximab maintenance (RM) for 2 years was compared with a metabolic/molecular response-adapted post-induction management in patients with advanced-stage FL who responded to induction immunochemotherapy [61]. At the end of induction (EOI) therapy, patients with partial response or complete response were randomly assigned to standard RM (12 doses of rituximab), only observation, or weekly rituximab dosing with residual disease monitoring (maximum 12 doses). Standard RM was significantly better than the metabolic/molecular response-adapted management in terms of PFS (3-year PFS: 86% vs. 72%; *p* < 0.001). The 3-year OS was not significantly different between the two groups (98% vs. 97%, respectively, *p* = 0.238). This report concluded that patients with FL who respond to induction immunochemotherapy should be offered 2 years of RM to lower the risk of lymphoma progression compared to response-adapted treatment.

However, Guerra et al. recently reported a secondary analysis of the trial, showing that the DS at EOI PET is a reliable and prognostic marker for PFS and OS in advanced-stage FL [62]. The 5-year PFS for patients with negative EOI PET (DS 1, 2, or 3) was 71%, compared to 36% for those with positive EOI PET (DS 4 or 5), with a hazard ratio (HR) of 3.49 (95% CI, 2.57 to 4.72). The 5-year OS was 96% in patients with negative EOI PET and 82% in those with positive EOI PET (*p* < 0.001).

In patients with diffuse large B-cell lymphoma (DLBCL), quantitative assessment of the change in standardized uptake value maximum (ΔSUVmax) after two cycles or four cycles of R-CHOP has shown predictive value compared to the visual assessment of DS. A meta-analysis of eight trials found that ΔSUVmax (>66% reduction after 2 cycles of R-CHOP and >70% reduction after 4 cycles) had higher discriminative power and predictive values than the DS 4–5 criteria. Furthermore, ΔSUVmax after four cycles of R-CHOP has better NPV and PPV than iPET after two cycles [63].

#### 2.4.3. Response Assessment in Immunotherapy

The Lugano classification was developed based on experience with traditional chemotherapeutic or chemoimmunotherapeutic regimens, primarily incorporating rituximab. However, with the increasing availability of biological agents with immune mechanisms, atypical responses are becoming more common and necessitate flexibility in interpreting response assessments. Atypical responses in cancer immunotherapy can be classified into four categories: pseudoprogression, hyperprogression, durable response, and dissociated response [64]. Pseudoprogression refers to a temporary increase in tumor size or number of lesions due to excessive immunological activation or a delayed immunologic response. Hyperprogression describes a rapid and severe worsening of the disease. A durable response indicates a continued positive response to immunotherapy even after discontinuation of the treatment. A dissociated response occurs when some tumor cells grow while others regress, similar to the mixed response seen after chemotherapy.

Occasionally, tumor flare can occur during the first 2 to 3 weeks of treatment, though this is seen in less than 10% of cases. Tumor flare is characterized by a rapid, often painful, self-limited increase in lymph node size, and it is frequently accompanied by fever, lymphocytosis, rash, and bone pain [65]. Tumor flare has been observed following treatment with immunomodulatory drugs such as lenalidomide [66]. Similarly, ICIs have shown flare reactions or delayed responses in several clinical trials with HL and NHL, despite their impressive outcomes. For instance, in the phase II trial of nivolumab and AVD for early-stage unfavorable HL, 7% of patients (7/101) did not achieve complete remission at the EOT PET, though none experienced subsequent disease progression [67].

To address these challenges, the lymphoma response to immunomodulatory therapy criteria (LYRIC) was proposed as a provisional modification of the Lugano criteria adapted for immune-based therapy [68]. In addition to the Lugano criteria, LYRIC introduces a new response category called indeterminate response (IR), which includes IR(1): increase in overall tumor burden (sum of the product of the diameters [SPD] of up to 6 measurable lesions) of ≥50% in the first 12 weeks of therapy without clinical deterioration; IR(2): new lesions or an increase in one or more existing lesions by ≥50%, but without overall progression (i.e., <50% increase) of overall tumor burden (SPD) at any time during the treatment; and IR(3): increase in FDG uptake of one or more lesions without an increase in lesion size or number. Patients with an indeterminate response are allowed to continue treatment and are required to undergo subsequent evaluation within 12 weeks to confirm or refute true disease progression.

The LYRIC was primarily based on experience with ICI therapy, but it could also be applied to other immunotherapies, such as bispecific antibodies or engineered T-cells, that exhibit similar atypical response patterns. However, the optimal timing for FDG PET during immunotherapy to monitor the treatment response remains uncertain. Further international collaborative efforts are necessary to establish a consensus on using the 2014 Lugano Classification and/or LYRIC for evaluating response and disease progression in immunotherapy.

Early FDG PET/CT response was suggested by Chen et al. to predict OS in relapsed or refractory HL treated with nivolumab [69]. FDG PET/CT was obtained at a median of 2.0 months (interquartile range, 1.7–3.7 months) after nivolumab initiation. Patients were classified into four response categories: complete metabolic response (CMR), partial metabolic response (PMR), no metabolic response (NMR), or progressive metabolic disease (PMD), using the 2014 Lugano Classification and the 2016 LYRIC criteria. The response classification was consistent between the 2014 Lugano Classification and LYRIC, as all progression events classified as IR by LYRIC were confirmed in subsequent evaluations. The 2-year OS probability differed significantly among patients with PMD (0.53), NMR or PMR (0.80), and CMR (1.00) in the overall population (*p* = 0.02, 45 patients). In a recent retrospective study, assessment by FDG PET at 1 month after CAR T-cell infusion in patients with relapsed or refractory large B-cell lymphoma has also been suggested as an important biomarker, with disease progression at iPET being associated with poor OS [70].

### 2.5. Artificial Intelligence and Radiomics

Traditional computer learning involved explicit instruction, which made it difficult for systems to adapt to tasks not previously taught. Machine learning (ML) is typically classified into supervised, unsupervised, and reinforcement learning. In supervised learning, a model is trained on a dataset where the correct output is labeled for each input. In unsupervised learning, the model is provided with data without labels. In reinforcement learning, the model learns from feedback in the form of positive and negative reinforcement to adjust its actions and improve performance over time [71].

Artificial neural networks (ANNs) are interconnected layers of processing units used to implement ML. An ANN is typically structured with one input layer, one or more hidden layers, and one output layer. Deep learning (DL) is a subset of ANN algorithms that includes multiple hidden (deep) layers. DL models can discover intricate structures within large datasets using the backpropagation algorithm, which adjusts internal parameters based on the representation computed in each layer from the previous layer. DL models are categorized into typical networks that handle vector (one-dimensional) inputs and convolutional neural networks (CNNs) that handle two-dimensional or three-dimensional inputs. CNNs, in particular, have proven valuable for segmentation and feature extraction from medical images and have been widely applied in disease detection, classification, and prognostication [6].

Recently, a major focus of PET in lymphoma using DL has been tumor segmentation [72,73]. Tumor segmentation with current semi-automatic software is time-consuming and shows high intra- and inter-observer variability, particularly in diseases like advanced lymphomas, which often present with widespread multiple lesions. Physiological FDG uptake and excretion, with inconsistent shape and localization in functioning organs such as the brain, heart, kidneys, and urinary bladder, pose challenges for the automatic segmentation of lymphoma lesions in PET [74]. Capobianco et al. reported that, in patients with DLBCL, TMTV automatically processed by the DL algorithm using CNN was significantly correlated with TMTV determined by experts (ρ = 0.76; *p* < 0.001) and yielded 85% classification accuracy, 80% sensitivity, and 88% specificity. Both TMTV by DL and TMTV by experts were predictive of PFS (HR, 2.3 and 2.6, respectively; *p* < 0.001) and OS (HR, 2.8 and 3.7, respectively; *p* < 0.001) [75]. Moreover, the U-Net architecture, a fully CNN-based approach, has been developed to detect and segment tumors more precisely. Sundar et al. suggested a program that generates fully automated, semantic multiple-organ segmentation from whole-body FDG PET/CT images using the state-of-the-art nnU-Net segmentation framework [76]. Leung et al. also reported a deep semisupervised transfer learning approach for fully automated whole-body tumor segmentation on PET/CT using nnU-Net architecture, which is used for tumor quantification and prognostic risk stratification [77].

Radiomics converts imaging data into high-dimensional quantitative features through advanced mathematical analysis. This approach is based on the assumption that medical images contain disease-specific information that is imperceptible through human visual inspection. As more medical imaging data became available in digital format, new sophisticated software applications were developed to analyze it [78].

Radiomics features in medical imaging are broadly categorized into semantic and agnostic features. Semantic features, such as size, shape, and location, describe geometric properties of the lesion. In contrast, agnostic features—such as histogram metrics (skewness, kurtosis), texture, and filter-based features—are mathematically extracted quantitative descriptors used to capture lesion heterogeneity. Agnostic features can be further divided into first-, second-, or higher-order statistical outputs. First-order features describe the distribution of intensities of individual voxels. Metrics such as mean, median, and mode describe the central tendency of the data, while percentiles, skewness, kurtosis, and entropy describe the symmetry and heterogeneity of the distribution. Second-order features, commonly known as texture features, describe statistical interrelationships between neighboring voxels with similar or dissimilar contrast values. For example, in coarse texture, neighboring pairs of voxels are likely to have similar gray levels, whereas in fine texture, neighboring voxel values are independent. Higher-order features apply filtering to the image to extract repetitive or non-repetitive patterns by emphasizing edges, different length scales, or varying gray levels [79,80].

Radiomics applications in lymphoma typically focus on either the classification of lymphoma subtypes or the prediction of clinical outcomes. de Jesus et al. investigated FDG PET/CT radiomics-based ML classifiers to differentiate FL from DLBCL [81]. Since FL and DLBCL cannot be distinguished by visual interpretation of FDG PET/CT, radiomics features were extracted from baseline FDG PET/CT and analyzed using ML algorithms (AdaBoost, Gradient Boosting, and XGBoost). The Gradient Boosting classifier achieved the best discrimination performance using 136 radiomics features (AUC = 0.86, accuracy = 80%). In comparison, the SUVmax-based logistic regression model was also evaluated (AUC = 0.79, accuracy = 70%). The Gradient Boosting classifier had a significantly greater AUC and accuracy than the SUVmax-based logistic regression model (*p* ≤ 0.01). Lippi et al. reported that texture analysis features extracted from FDG PET, combined with multiple-instance ML algorithms, can distinguish HL from four other types of malignant lymphomas (DLBCL, FL, HL, and MCL) [82]. From the phase III trial (N = 1418), which was originally designed to compare the use of R-CHOP with obinutuzumab plus CHOP in patients with previously untreated DLBCL, FDG PET radiomics features provided improved prognostic predictions for survival. The random forest model demonstrated a clearer distinction of high-risk populations compared to the International Prognostic Index [83].

Although radiomics features have shown potential for various purposes in numerous studies, proper validation and clinical evidence for their efficacy are still limited. The lack of standardization in methodology has resulted in poor reproducibility and interchangeability. Standardization and harmonization of image acquisition, processing, and radiomics feature extraction are essential. Furthermore, radiomic models should be tested on external data not used for their development or, if not possible, validated using appropriate internal techniques [84].

## 3. FAPI PET

### 3.1. Diagnosis

FAPI-based radiotracers are anticipated to become the next generation of pan-cancer imaging agents for PET. Since tumors require a supporting stroma, which can be larger in volume than the neoplastic cells themselves, FAPI PET is particularly useful for tumors with a strong desmoplastic reaction, such as breast, colon, and pancreatic cancers, as well as peritoneal carcinomatosis. Additionally, small primary or metastatic lesions in organs like the brain, liver, pancreas, and gastrointestinal tract can be detected due to the low physiological background uptake of FAPI. FAPI PET offers higher target-to-background ratios and sharper image contrast compared to FDG PET. However, inflammation, bone degeneration, scars, and healing areas can also express FAP and cause non-specific FAPI accumulation during tumor detection [85].

Hirmas et al. compared the diagnostic accuracy of ^68^Ga-FAPI PET and FDG PET in 115 patients with various malignancies [86]. ^68^Ga-FAPI PET demonstrated higher diagnostic accuracy than FDG PET for colorectal and prostate cancers. It was comparable to FDG PET in diagnosing breast cancer and head and neck cancers. However, ^68^Ga-FAPI PET showed lower accuracy than FDG PET for urinary bladder cancer, kidney cancer, lymphoma, and myeloma. Among the 10 patients with lymphoma, ^68^Ga-FAPI PET showed lower accuracy (88.9% vs. 100%), sensitivity (87.5% vs. 100%), and NPV (50% vs. 100%) compared to FDG PET at the per-patient level. At the per-region level, ^68^Ga-FAPI PET also demonstrated lower accuracy (90% vs. 96.7%), sensitivity (78.6% vs. 100%), and NPV (84.2% vs. 100%) than FDG PET.

**Figure 3:**
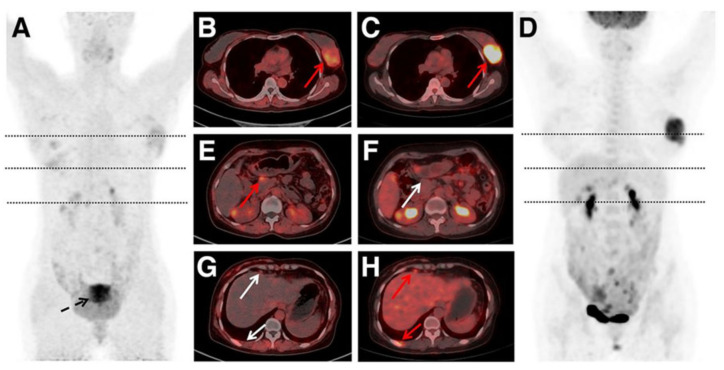
Images of ^68^Ga-FAPI and FDG PET/CT in a 46-year-old woman with lymphoblastic leukemia/lymphoma. (**A**) Maximum-intensity projection of ^68^Ga-FAPI PET. (**B**–**H**) Axial ^68^Ga-FAPI PET/CT images (**B**,**E**,**G**) and ^18^F-FDG PET/CT images (**C**,**F**,**H**). (**D**) Maximum-intensity projection of FDG PET. Left breast involvement was detected with intense ^68^Ga-FAPI and FDG uptake (**B**,**C**, arrows). PET/CT image showed lesion with focal ^68^Ga-FAPI uptake in pancreas (**E**, arrow) and normal FDG activity (**F**, arrow). FDG PET/CT image showed lymph nodes positive for uptake in internal mammary and subpleural areas (**H**, arrows), without corresponding ^68^Ga-FAPI uptake (**G**, arrows). Intense ^68^Ga-FAPI uptake was noted in uterus (**A**, arrow). (Figure from [87]. Copyright © Society of Nuclear Medicine and Molecular Imaging).

Chen et al. reported a study involving 162 patients with NHL and 24 with HL to compare the diagnostic performance of ^68^Ga-FAPI PET/CT and FDG PET/CT [87]. FDG PET/CT demonstrated higher staging accuracy than ^68^Ga-FAPI PET/CT (98.4% vs. 86.0%). Among 5980 lymphoma lesions, FDG PET/CT detected more nodal (4624 vs. 2196) and extranodal (1304 vs. 845) lesions than ^68^Ga-FAPI PET/CT. There were 52 lesions that were positive on ^68^Ga-FAPI but negative on FDG, and 2939 lesions that were positive on FDG but negative on ^68^Ga-FAPI. Immunohistochemistry staining results showed that hexokinase 2 (HK2) and glucose transporter 1 (GLUT1) cell densities were significantly higher than FAP in most lymphoma subtypes (*p* < 0.001). However, FAP was expressed in some lymphomas without HK2 and/or GLUT1. These findings suggest that ^68^Ga-FAPI PET may complement FDG PET in diagnosing, staging, and assessing treatment response in indolent lymphoma or lymphoma with low FDG avidity. A representative case comparing ^68^Ga-FAPI PET and FDG PET is shown in Figure 3 [87].

### 3.2. FAP-Targeted CAR-T Cell Therapy

Despite the recent dramatic advancements in CD19 CAR-T cell therapy for hematologic malignancies, its application in solid tumors remains limited. Most tumor antigens are also expressed at low levels on some normal or non-malignant tissues, compromising CAR-T cell target recognition and increasing the likelihood of toxicity. Additionally, tumor cells exhibit various levels of antigen expression or even a lack of expression, leading to antigen heterogeneity within the tumor. Finally, target antigens may be masked by the TME, reducing the efficacy of CAR-T cell therapy [88].

One strategy for treating solid tumors involves targeting non-cancerous cells that modulate the TME. CAFs are potential candidates for this approach, leading to the development of FAP-targeted CAR-T cells [89]. In this context, FAPI PET has the potential to measure FAP expression and guide FAP CAR-T cell therapy as an imaging biomarker. Lee et al. reported that ^18^F-aluminum (Al)F-FAPI-74 PET/CT imaging is useful in monitoring the therapeutic response to FAP CAR-T cell therapy in mouse models of lung cancer [90]. Consequently, the role of FAPI PET in relapsed or refractory lymphomas is also expected to be explored [91].

## 4. ImmunoPET

### 4.1. CD20

CD20 is a B-cell surface antigen overexpressed in most B-cell lymphomas. It functions as a transmembrane calcium channel, mediating B-cell proliferation and differentiation. Rituximab is the most commonly used mAb for treating CD20-positive lymphomas [92]. Jauw et al. reported that the tumor uptake of ^89^Zr-rituximab in PET imaging was concordant with CD20 expression in tumor biopsies in five out of six patients [93]. Furthermore, the tumor targeting and radiation dose of RIT with ^90^Y-rituximab in CD20-positive B-cell lymphoma were predicted by ^89^Zr-rituximab PET in a study involving five patients [94].

There were two commercially available RITs: ^131^I-labeled tositumomab (Bexxar^®^) and ^90^Y-ibritumomab tiuxetan (Zevalin^®^), both used for relapsed or refractory NHL. ^131^I-labeled tositumomab is a murine antibody targeting CD20, while ^90^Y-ibritumomab tiuxetan is a murine variant of rituximab. Despite the efficacy and acceptable safety profiles, their use did not gain widespread adoption in clinical practice, partially due to concerns about cost-effectiveness and the complexity of the delivery process. Recently, there has been growing interest in exploring other therapeutic radionuclides, such as the β-emitter ^177^Lu or α-emitters like ^211^At, ^212^Pb, ^213^Bi, and ^227^Th [95].

### 4.2. CAR-T Cell

CD19 is physiologically expressed during B-cell differentiation and is therefore present in most B-cell lymphomas. Currently approved CAR-T cell therapies target CD19 on B-cell lymphomas [96]. PET imaging is an attractive strategy for non-invasive, longitudinal monitoring of CAR-T cell distribution in vivo. However, data on PET imaging for CD19-positive cells remain limited.

Recently, inducible T-cell co-stimulator (ICOS or CD278), a co-stimulatory molecule upregulated during T-cell activation, was proposed as a candidate target for CAR-T cell monitoring using immunoPET. Simonetta et al. reported that ^89^Zr-desferrioxamine (DFO)-ICOS mAb PET/CT imaging successfully visualized murine CD19.28z CAR-T cells during antitumor responses in B-cell lymphoma [97]. Minn et al. suggested the transduction of CAR-T cells with prostate-specific membrane antigen (PSMA) and their tracking using 2-(3-{1-carboxy-5-[(6-^18^F-fluoro-pyridine-3-carbonyl)-amino]-pentyl}-ureido)-pentanedioic acid (^18^F-DCFPyL)-PET. CD19-tPSMA(N9del) CAR-T cells were tracked with ^18^F-DCFPyL PET in a Nalm6 model of acute lymphoblastic leukemia [98].

### 4.3. PD-1/PD-L1

Immune checkpoint proteins, such as programmed cell death protein 1 (PD-1) on T cells and programmed cell death-1 ligand (PD-L1) on tumor cells, bind to each other and prevent the immune response from killing tumor cells. However, ICIs that block the binding of PD-1 or PD-L1 allow T cells to destroy tumor cells [99]. PET radiotracers targeting PD-1 or PD-L1 have been investigated for advanced non-small cell lung cancer (NSCLC) and melanoma. Uptake of ^18^F-labeled anti-PD-L1 adnectin (^18^F-BMS-986192) and ^89^Zr-nivolumab in patients with NSCLC was correlated with PD-(L)1 expression by immunohistochemistry in tumor tissues [100]. In a pilot study involving 10 patients with metastatic melanoma who underwent ^18^F-BMS-986192 PET before and during ICI therapy, heterogeneous uptake of ^18^F-BMS-986192 was observed in all metastatic lesions. Moreover, high baseline ^18^F-BMS-986192 uptake in lesions was correlated with a positive response to ICI treatment [101]. ^89^Zr-atezolizumab imaging was performed in 22 patients with locally advanced or metastatic bladder cancer, NSCLC, or triple-negative breast cancer before starting atezolizumab therapy. Clinical responses in these patients correlated better with pretreatment PET uptake than with predictive biomarkers from immunohistochemistry or RNA sequencing [102]. ICIs have also been successful in certain lymphoma subtypes, such as classic HL and primary mediastinal B-cell lymphoma [103]. Since ICIs are expected to play an increasingly important role in lymphoma therapy, further research is needed to explore the potential role of PD-(L)1-targeted PET in lymphoma.

### 4.4. CD30

CD30 is physiologically expressed on a small subset of B cells and T cells and is a member of the tumor necrosis factor receptor superfamily. It is also expressed in certain hematologic malignancies, including anaplastic large-cell lymphoma and HL [104]. Brentuximab vedotin, an antibody-drug conjugate targeting CD30, has become an important component of therapy for relapsed or refractory CD30-positive lymphomas [105]. ^89^Zr-DFO-anti-CD30 mAb (AC-10) has shown specific binding to CD30 both in vitro and in vivo. ^89^Zr-DFO-AC-10 PET demonstrated high tumor-to-background tissue contrast in mice models bearing subcutaneous CD30-positive lymphoma [106]. However, no in-human studies have been conducted so far.

### 4.5. CD8

CD8 is physiologically expressed on activated cytotoxic T cells and in T-cell lymphomas such as cutaneous T-cell lymphoma and PTCL. Cytotoxic CD8-positive T cells are a crucial part of the immune response to cancer. High levels of tumor-infiltrating cytotoxic T cells have frequently been associated with better outcomes in patients with various cancers treated with ICIs [107]. A phase I first-in-humans study of ^89^Zr-IAB22M2C (anti-CD8 minibody) was conducted to evaluate CD8-targeted PET imaging of tumor-infiltrating T cells in 15 patients with metastatic melanoma, non-small cell lung cancer, and hepatocellular carcinoma [108]. Although the study did not include patients with lymphoma, ^89^Zr-IAB22M2C accumulated in tumors and CD8-rich tissues (e.g., spleen, bone marrow, lymph nodes) and correlated with the response following immunotherapy.

### 4.6. CXCR4

**Figure 4:**
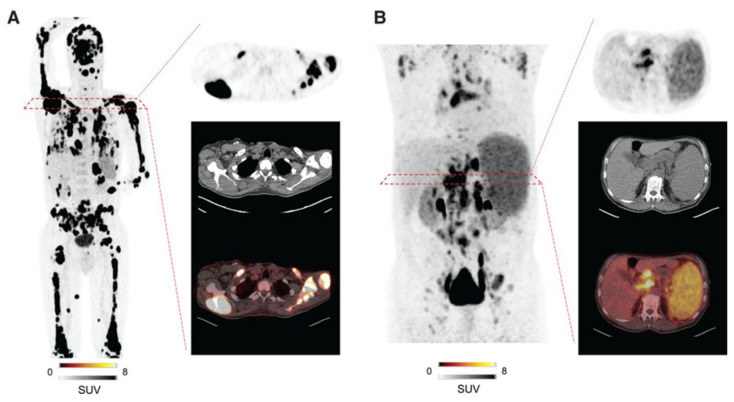
Maximum-intensity projections of patients with hematologic malignancies imaged with CXCR4-directed ^68^Ga-Pentixafor. Target lesion is also displayed on transaxial PET, CT, and PET/CT. Patient diagnosed with multiple myeloma (**A**; SUVmax in target lesion, 74.3) and MCL (**B**; SUVmax in target lesion, 17.2). Substantially low background activity allowed for precise determination of disease sites. (Figure from [111]. Copyright © Society of Nuclear Medicine and Molecular Imaging).

C-X-C Motif Chemokine Receptor 4 (CXCR4) is a seven-transmembrane G protein-coupled receptor belonging to the chemokine receptor family, playing a crucial role in cell communication, proliferation, and migration. While CXCR4 is physiologically expressed by numerous hematological and immune cells, its overexpression is well documented in hematological malignancies and various lymphomas. ^68^Ga-C_60_H_77_GaN_14_O_14_ or CPCR4.2 (Pentixafor) PET has been shown to visualize lymphoplasmacytic lymphoma, chronic lymphocytic leukemia, MCL, MZL, and central nervous system lymphoma, with particularly high uptake observed in MCL and MZL [109]. Therefore, ^68^Ga-Pentixafor PET could be especially useful in lymphomas with low FDG avidity. Furthermore, a theranostic option using ^177^Lu/^90^Y-Pentixather has also been proposed. Dreher et al. reported that 21 heavily pretreated patients with hematological malignancies underwent CXCR4-directed radioligand therapy using ^90^Y-Pentixather following CXCR4-directed ^68^Ga-Pentixafor PET/CT. This therapy effectively mediated myeloablation, allowing for hematopoietic stem cell transplantation [110]. Representative cases of CXCR4-directed ^68^Ga-Pentixafor PET are shown in Figure 4 [111].

## 5. Future Directions

The future of PET imaging in lymphoma is promising, driven by technological advancements and the development of new radiotracers. With AI-based tools, PET data analysis using quantitative imaging biomarkers is expected to enable precise prognostication and treatment response prediction. The exploration of the TME using FAPI PET will likely expand the role of PET in non-FDG-avid lymphomas. ImmunoPET, with its ability to image disease-specific antigens, could become a crucial tool in developing and optimizing immunotherapies, allowing for real-time tracking of CAR-T cells and other immune-modulating treatments. Further clinical trials and studies on FAPI PET and immunoPET are necessary to validate their clinical utility and optimize patient selection for targeted therapies. Several current clinical trials (recruiting and not yet recruiting) on lymphoma using PET are listed in Table 2.

RIT holds great potential for advancing lymphoma treatment and is an important future direction for PET imaging. As immunoPET evolves, it can aid in the development and optimization of RIT by providing critical pharmacokinetic and pharmacodynamic information. A promising area of research involves radiolabeled antibodies targeting specific lymphoma-associated antigens, such as CD20, CD30, and CD19, labeled with β- or α-emitting radionuclides for therapeutic purposes. By linking PET imaging with RIT, clinicians can non-invasively evaluate antigen expression and distribution before treatment, ensuring optimal targeting and predicting therapeutic response. Furthermore, early identification of responders to RIT through immunoPET allows real-time monitoring of treatment efficacy, enabling adjustments to the therapeutic regimen. This approach could help personalize RIT by selecting patients whose tumors express high levels of the target antigen, improving treatment efficacy and reducing toxicity.

Additionally, combining RIT with other treatment modalities, such as chemotherapy, CAR-T cell therapy, or ICIs, presents an exciting opportunity for synergy. For instance, combining RIT with ICIs could enhance the anti-tumor immune response by amplifying the effects of radiation on both tumor cells and the immune system. Further research is needed to explore these combinations and their impact on treatment outcomes. Future studies should also focus on improving the safety profile of RIT by optimizing radionuclide selection and dosimetry. RIT has the potential to become an integral part of lymphoma treatment, particularly for patients with relapsed or refractory disease.

## 6. Conclusions

PET imaging has significantly advanced the management of lymphoma, with FDG PET playing a pivotal role in diagnosis, staging, prognostication, and treatment response evaluation. However, the limitations of FDG have spurred the development of novel tracers, such as FAP-targeted and immunoPET tracers, which offer more specific insights into lymphoma biology and immune interactions. As new PET tracers and imaging methodologies are integrated into clinical practice, they promise to enhance the precision of lymphoma diagnosis and therapy, particularly in the era of immunotherapy. Continued research into AI-driven PET analysis, alongside validation of emerging tracers, will be essential for optimizing patient outcomes and furthering the potential of PET imaging in lymphoma.

## Figures and Tables

**Table 1:** FDG avidity in the main subtypes of lymphoma [15,16,17,18,19,20,21].

Histology	FDG Uptake
Hodgkin lymphoma	High
Diffuse large B-cell lymphoma	High
Follicular lymphoma	
Grade III	High
Grade I and II	Moderate
Marginal zone lymphoma	
Gastric mucosa-associated lymphoid tissue	Low
Non-gastric mucosa-associated lymphoid tissue	Moderate
Primary cutaneous marginal zone lymphoma	Very low
Nodal marginal zone lymphoma	Moderate
Splenic marginal zone lymphoma	Low to high
Mantle cell lymphoma	
Nodal	High
Gastrointestinal or bone marrow	Low
Chronic lymphocytic leukemia/small lymphocytic lymphoma	Low to moderate
Peripheral T-cell lymphoma	Low to high
Cutaneous T-cell lymphoma	
Mycosis fungoides	Low to moderate
Sezary syndrome	Low to moderate

**Table 2:** Current clinical trials (recruiting and not yet recruiting) on lymphoma using PET.

ID	Title	Summary	Study Start
NCT06125028	^68^Ga-Pentixafor PET Imaging for Staging of Marginal Zone Lymphoma (LYMFOR)	A pivotal phase 3 clinical trial to assess the diagnostic performance and safety of ^68^Ga-Pentixafor vs. FDG PET/CT for staging of patients with confirmed MZL, exemplary for CXCR4-positive malignant lymphomas	2024-6
NCT06148220	A Study of the Clinical Application of ^18^F-RCCB6 and ^68^Ga-NOTA-RCCB6 PET/CT Imaging in the Diagnosis of CD70-expressing Multiple Tumors	To establish and optimize the imaging method, physiological and pathological distributions, and diagnostic accuracy in renal cell cancer (especially clear cell carcinoma) and lymphoma	2023-10-1
NCT05390814	^18^F-Fludarabine PET/MRI in Primary CNS Lymphoma (FLUDALOC)	To characterize the cerebral distribution and ^18^F-Fludarabine uptake in newly diagnosed primary CNS lymphomas before surgery, chemotherapy or radiotherapy, using PET/MRI	2023-12-18
NCT06461182	^68^Ga-CXCR4 PET/CT in Indolent B-cell Lymphoma (PentixaFor)	To explore the efficacy of ^68^Ga-PentixaFor PET/CT in detecting, assessing treatment response, and monitoring the risk of aggressiveness in indolent B-cell lymphoma	2024-4-29
NCT05371132	Study to Evaluate CD8 PET Imaging as a Marker of Immune Response to Stereotactic Body Radiation Therapy (ELIXR)	Phase I trial to investigate the effect of radiation therapy on the immune system, specifically CD8-positive T cells, in lymphoma patients receiving bridging radiation therapy before CAR T-cell infusion, and metastatic patients with solid tumor malignancies receiving SBRT	2022-6-20
NCT06522932	PET Imaging Study of ^64^Cu-GRIP B for Patients Receiving CD19-directed CAR-T Therapy	To establish the feasibility of granzyme B detection with ^64^Cu-GRIP B PET in participants with relapsed/refractory NHL receiving CD19-directed CAR-T cell therapy	2024-8-30
NCT05096234	^18^F-F-AraG PET Imaging to Evaluate Immunological Response to CAR T Cell Therapy in Lymphoma	To explore the relationship of change in ^18^F-AraG PET signal following CAR T cell treatment with changes in T cell infiltration in tumor biopsies	2021-9-28
NCT05404048	PD-L1 PET-imaging During CAR T-cell Therapy	A single-center, single-arm pilot trial designed to evaluate the expression of PD-L1 in patients with Large B-cell lymphoma and its role in non-responsiveness to CAR T-cell therapy in a non-invasive manner. Moreover, within this trial, ^89^Zr-atezolizumab PET/CT as a tool to distinguish lymphoma activity from a treatment-related inflammatory activity in patients with an end-of-treatment positive FDG PET/CT	2022-5-18
NCT06636175	^64^Cu-LLP2A for Imaging Hematologic Malignancies	Early phase I evaluation of ^64^Cu-LLP2A for imaging hematologic malignancies including multiple myeloma and low-grand lymphoma	2024-11-30
NCT06224309	Preliminary Assessment of ^18^F-BL40 in PET/CT scans	To evaluate the diagnostic utility of ^18^F-BL40 PET/CT to stage patients with CXCR4-expressing tumors	2024-5
NCT04169321	Granzyme B PET Imaging Drug as a Predictor of Immunotherapy Response to Checkpoint Inhibitors	First in Human study of ^68^Ga-NOTA-hGZP PET imaging to test the safety and effectiveness in subjects with cancer undergoing treatment with a checkpoint inhibitor	2020-6-16
NCT05627115	Response Adapted Incorporation of Tislelizumab into the Front-line Treatment of Older Patients With Hodgkin lYmphoma (RATiFY)	To test the effect of initial treatment of tislelizumab (PD-1 antibody) for Hodgkin lymphoma in patients aged 60 years and older guided by PET-response assessment	2024-3-1
NCT05004961	The Performance of Multi-tracer Multimodality PET in Lymphoma	To evaluate nodal and extranodal lymphoma lesions uptake by different molecular probes such as FDG, FAPI-04 via multimodality PET (PET/CT and PET/MR)	2019-9-15

## Data Availability

No new data were created or analyzed in this study. Data sharing is not applicable to this article.

## Abbreviations

ABVD	doxorubicin, bleomycin, vinblastine, and dacarbazine
AI	artificial intelligence
ANN	artificial neural network
AVD	doxorubicin, vinblastine, and dacarbazine
BEACOPP-14	repeated bleomycin, etoposide, doxorubicin, cyclophosphamide, vincristine, procarbazine, and prednisone every 14 days
BM	bone marrow
BV	brentuximab vedotin
CAF	cancer-associated fibroblast
CAR T-cell	chimeric antigen receptor T-cell
CMR	complete metabolic response
CNN	convolutional neural network
CT	computed tomography
CXCR4	C-X-C Motif Chemokine Receptor 4
DFO	desferrioxamine
DL	deep learning
DLBCL	diffuse large B-cell lymphoma
Dmax	maximum tumor dissemination
DS	Deauville score
eBEACOPP	escalated doses of bleomycin, etoposide, doxorubicin, cyclophosphamide, vincristine, procarbazine, and prednisone
EOI	end of induction
EOT PET	end-of-treatment PET
FAP	fibroblast activation protein
FAPI	fibroblast activation protein inhibitor
FDG PET	^18^F-fluorodeoxyglucose positron emission tomography (FDG PET)
18F-DCFPyL	2-(3-{1-carboxy-5-[(6-18F-fluoro-pyridine-3-carbonyl)-amino]-pentyl}-ureido)-pentanedioic acidCRS = cytokine release syndrome
FL	follicular lymphoma
GLUT1	glucose transporter 1
HK2	that hexokinase 2
HL	Hodgkin lymphoma
HR	hazard ratio
ICI	immune checkpoint inhibitor
ICOS	inducible T-cell co-stimulator
IFRT	involved-field radiation therapy
INRT	involved-node radiotherapy
iPET	interim PET
IR	indeterminate response
LYRIC	lymphoma response to immunomodulatory therapy criteria
mAb	monoclonal antibody
MCL	mantle cell lymphoma
ML	machine learning
MTV	metabolic tumor volume
MZL	marginal zone lymphoma
NHL	non-Hodgkin lymphoma
NK	natural killer
NMR	no metabolic response
NPV	negative predictive value
OS	overall survival
PD-1	programmed cell death protein 1
PD-L1	programmed cell death-1 ligand
PFS	progression-free survival
PMD	progressive metabolic disease
PMR	partial metabolic response
PPV	positive predictive value
PTCL	peripheral T-cell lymphoma
RAPID	Randomized Phase III Trial to Determine the Role of FDG PET Imaging in Clinical Stages IA/IIA Hodgkin’s Disease
RATHL	Response-Adjusted Therapy for Advanced Hodgkin Lymphoma
R-CHOP	rituximab, cyclophosphamide, doxorubicin, vincristine, and prednisone
RIT	radioimmunotherapy
RM	Rituximab maintenance
ROI	region of interest
SPD	sum of the product of the diameters
SUA	standardized uptake value normalized by injected dose of radioactivity with body surface area
SUL	standardized uptake value normalized by injected dose of radioactivity with lean body mass
SUV	standardized uptake value
TLG	total lesion glycolysis
TME	tumor microenvironment
